# 

**Published:** 1860-10

**Authors:** 


### CONTENTS.

ORIGINAL ESSAYS AND COMMUNICATIONS.

Dental Teachings. By W. H. Atkinson ........ 185
The Anatomy, Physiology, Pathology, and Remedial Treatment of the Fifth Pair of Nerves. By J. H. M'Quillen, D. D. S. ........ 189
Exposed Pulps,—Secondary Dentine. By Geo. Watt ........ 204
The Oneness of Dentistry ........ 210
Slabs from the Furnace. By Joe Stackpole ........ 213
A Critic Criticised ........ 218

PROCEEDINGS OF SOCIETIES.

Proceedings of the American Dental Association ........ 222
Selections ........ 237
Editorial ........ 244

Communications must reach the Editor as early as the 15th, to insure insertion in the next issue.

